# **ADAPT: The Wrong Way to Stop a Clinical Trial**

**DOI:** 10.1371/journal.pctr.0010035

**Published:** 2006-11-17

**Authors:** Steven E Nissen

In an accompanying article published in *PLoS Clinical Trials*, the Alzheimer's Disease Anti-Inflammatory Prevention Trial (ADAPT) Research Group report the cardiovascular outcomes from their study [1]. The circumstances surrounding the termination of ADAPT were unusual and provide an important lesson for all clinical trialists, demonstrating the importance of following rigorous procedures for prematurely stopping clinical trials. In this case, stopping the trial before its intended completion resulted in data that cannot be reliably interpreted.

On September 30, 2004, Merck withdrew rofecoxib (Vioxx) from the market after the trial's data safety and monitoring board (DSMB) recommended termination of a placebo-controlled study of this agent in the prevention of colon polyps [2]. The reason for study termination was a statistically significant increase in adverse cardiovascular outcomes. On December 17, 2004, Pfizer announced that termination of a trial of celecoxib (Celebrex) in colon polyp prevention, because it also showed statistically significant evidence for increased cardiovascular event rates [3]. The results of these two trials were subsequently published in February 2005 in the *New England Journal of Medicine* [4,5]. The revelations about increased cardiovascular events with these “coxibs” generated enormous public attention and concern. However, in both cases, the decision-making leading to trial discontinuation was handled appropriately through the regular reviews conducted by an independent DSMB.

Three days following the announcement of the termination of the celecoxib colon polyp prevention study, the National Institute of Health (NIH), issued a press release entitled “Use of Non-Steroid Anti-Inflammatory Drugs Suspended in Large Alzheimer's Disease Prevention Trial” [6]. The NIH press release stated that “data from the ADAPT trial indicated an apparent increase in cardiovascular and cerebrovascular events among participants taking naproxen when compared to placebo.” In the press release, NIH Director Dr. Elias Zerhouni stated that “this step is being taken as a precautionary measure to ensure the safety of the study's participants” and that “the investigators made their decision based on the risk/benefit analysis specific to this trial.” Shortly following the NIH announcement, the Food and Drug Administration (FDA) issued a public statement that “based on emerging information from a long-term prevention trial, the risk of cardiovascular and cerebrovascular events may increase among patients taking naproxen” [7].

These announcements generated front-page headlines such as “Heart Risk Seen in Naproxen” (*Wall Street Journal*), “Tough Choice: Pain or Risk” (*USA Today*), and “Patients, Doctors Agonize over Risks of Painkiller (*Los Angeles Times*) [8]. Occurring immediately following the revelations about rofecoxib and celecoxib, the naproxen announcement generated considerable public apprehension [8]. Physicians received many urgent calls from worried patients. However, there was a major problem with the naproxen warning: it was not based upon the application of standard procedures for stopping an ongoing clinical trial. During the subsequent FDA hearings to set policy on nonsteroidal anti-inflammatory agents (NSAIDs) and COX-2 inhibitors, I described the warnings about naproxen as “the medical equivalent of yelling ‘fire' in a crowded auditorium” [9].

## The Data Finally Become Available

Now, nearly two years after the closure of the ADAPT study, we finally get to see the data that resulted in the public warning about naproxen [1]. For the standard composite endpoint of cardiovascular death, myocardial infarction, and stroke, there were 17 events in celecoxib treatment group, 23 in the naproxen arm, and 22 in the placebo. The hazard ratio for naproxen compared with placebo was 1.57 with 95% confidence intervals of 0.87 to 2.81, *p* = 0.13. A broader composite outcome that added heart failure and transient ischemic attack yielded a marginally significant *p* value when comparing naproxen with placebo.

We must ask whether a DSMB would stop an ongoing clinical trial for such findings. The answer, it appears, was that the DSMB did not stop the trial; NIH officials did [8]. There was no regularly scheduled DSMB safety review that resulted in study termination. It appears that NIH officials, concerned about revelations regarding the safety of coxibs in the colon polyps studies, simply decided to look at the cardiovascular event results in ADAPT [8,10]). Seeing a marginally significant difference between naproxen and placebo, they abruptly stopped the trial. Indeed, NIH officials went even further and issued the public warning [6].

Both the termination of this trial and the issuance of a public warning about naproxen were inappropriate and reflected faulty logic. These actions unnecessarily created public consternation and denied the participants in the trial the opportunity for their commitment to result in any benefit to themselves or society [11].

## Reinforcing Rigorous Methods for Safety Monitoring of Ongoing Trials

Current NIH policies may have contributed to the decision to prematurely terminate ADAPT. Surprisingly, NIH rules allow the leadership of various scientific divisions to have unblinded access to data. Even more surprisingly, principal investigators are also often unblinded. This policy provides for considerable temptation to interfere with trial management. It is well recognized that during the conduct of randomized trials that hazard ratios are often unstable, sometimes drifting over time into marginal levels of “significance.” This is an artifact of statistics. If you repeatedly sample data, the multiplicity of “looks” at the data ensure that there will occasionally appear a transient “signal” of benefit or harm. These “signals” are not reliable.

We have many sources of evidence that refute the notion that naproxen increases the risk of adverse cardiovascular outcomes. A recent meta-analysis by McGettigan and Henry [12] examined the relative cardiovascular risks for 23 studies of NSAIDs and COX-2 inhibitors. The relative risk for naproxen was 0.97 with 95% confidence intervals ranging from 0.87 to 1.07. The FDA Advisory Panel meeting in February 2005 opined that naproxen was the most appropriate comparator with which to evaluate the relative risks of new anti-inflammatory agents [9].

To avoid inappropriate premature termination of trials, statisticians and clinical trialists long ago adopted rigorous “stopping rules” for safety monitoring of ongoing trials [13–17]. These rules require a much higher level of significance for harm or benefit earlier in the trial. The most common approach, originally described by O'Brien and Fleming [13], would have precluded early stopping of ADAPT and avoided the generation of spurious findings. Strict investigator discipline and appropriate prespecification of stopping rules are required to enforce this type of rigorous approach.

Who shares responsibility for the improper termination of ADAPT? In my opinion, a major factor was the inappropriate unblinding of the trial by leadership at the NIH. Allowing unrestricted access to the study data by NIH officials represents an unwise policy and can only lead to errors of this kind.

The circumstances surrounding the termination of ADAPT demonstrate the importance of following rigorous procedures for prematurely stopping clinical trials.

Futhermore, the principal investigator and the Steering Committee should have, when approached by NIH officials about premature examination of the trial data, said “no.” Similarly, the DSMB should have resisted efforts to improperly interrupt an ongoing trial. If rebuffed by NIH officials, both the Steering Committee and the DSMB should have publicly disavowed the decision to terminate the trial or, alternatively, explained that the reasons for termination did not include the finding of a hazard for naproxen treatment.

Management of clinical trials is a major public responsibility. It takes self-discipline and a precise understanding of statistical methods. For ADAPT, accepted scientific procedures were not followed, resulting in an inappropriate public warning. Accordingly, the trial results cannot be reliably interpreted.

## Abbreviations

ADAPT: Alzheimer's Disease Anti-Inflammatory Prevention Trial
DSMB: data safety and monitoring board
FDA: Food and Drug Administration
NIH: National Institutes of Health
NSAID: nonsteroidal anti-inflammatory agent
